# COVID-Somnia: A Multicentric Study on Sleep Disturbances During the COVID-19 Pandemic With Spatial Mapping of Hotspots

**DOI:** 10.7759/cureus.39213

**Published:** 2023-05-19

**Authors:** Neeraj Pawar, Anindo Majumdar, Nisanth M Nedungalaparambil, Lakshmi Nair, Jasimudeen Sulaiman, Suruchi Gupta, Katie J Shillington, Victor Ng, Rajee Reghunath, Jennifer D Irwin

**Affiliations:** 1 Community and Family Medicine, All India Institute of Medical Sciences, Rae Bareli, Rae Bareli, IND; 2 Community and Family Medicine, All India Institute of Medical Sciences, Bhopal, Bhopal, IND; 3 Department of Emergency Medicine, Amala Institute of Medical Sciences, Thrissur, IND; 4 Department of Nursing, Amala College of Nursing, Thrissur, IND; 5 Department of Library, St. Stephen's College, Uzhavoor, Kottayam, IND; 6 Bloomberg School of Public Health, Johns Hopkins University, Baltimore, USA; 7 Faculty of Health Sciences, The University of Western Ontario, Ontario, CAN; 8 Schulich School of Medicine and Dentistry, The University of Western Ontario, Ontario, CAN

**Keywords:** india, psqi, pandemic, adults, sleep, covid-19

## Abstract

Objective

The purpose of this study was to document sleep quality and assess its sociodemographic, behavioral (i.e., tobacco use, alcohol use, and screen time), and mental-health-related indicators (i.e., anxiety and depression) in adults aged 30-59 years across three states of India, and to geo-locate state and district-level findings of sleep quality during the ongoing coronavirus disease 2019 (COVID-19) pandemic.

Methods

From October 2020 to April 2021, residents (aged 30-59 years) of Kerala, Madhya Pradesh, and Delhi completed a web-based survey that included sociodemographic and behavioral factors, clinical history of COVID-19, and mental health screening instruments for anxiety and depression, namely the Generalized Anxiety Disorder 2-item (GAD-2) and Patient Health Questionnaire-2 (PHQ-2). The Pittsburgh Sleep Quality Index (PSQI) was used to evaluate the quality of sleep. Average PSQI scores were geo-mapped.

Results

Of the 694 participants who responded, 647 completed the PSQI. The mean (SD) global PSQI score was 5.99 (3.2), with approximately 54% of participants reporting poor sleep quality (PSQI Score>5). Eight hotspot districts with severe sleep disturbances (mean score PSQI>6.5) were identified. Multivariable logistic regression analysis showed that compared to Madhya Pradesh, participants from Kerala and Delhi had 62% and 33% lower chances of having poor sleep quality, respectively. Those who screened positive for anxiety had higher odds of having poor sleep quality (adjusted odds ratio {aOR}=2.4, P=0.006*).

Conclusion

Overall, sleep quality was poor during the early stages of the COVID-19 pandemic (October 2020-April 2021), especially among those who reported high levels of anxiety. Among the three included states, there were differences in sleep quality.

## Introduction

In January 2020, the World Health Organization (WHO) declared the coronavirus disease 2019 (COVID-19) outbreak a Public Health Emergency of International Concern. To date, the COVID-19 pandemic has affected more than 764 million people globally, with reported deaths of over 6.9 million [1].

The transmissibility of this disease has forced nations to impose stringent protective measures such as the use of facemasks, social distancing, travel restrictions, and partial or complete lockdowns [2]. The government of India, in March 2020, enforced a nationwide lockdown restricting the free movement in public places of more than 1.3 billion people [3]. This decision helped in containing the spread of the virus and the overall mortality of the population, but also resulted in significant lifestyle changes among people who had to adapt to a completely new way of life rapidly; feelings of apprehension, fear, and stress about the pandemic, disruptions to social connections, and changes in daily routine affected citizens’ physical and mental health [4]. Stressful situations have been previously linked to sleep disturbances [5].

Adequate sleep is vital to the proper functioning of the human body and mind. In fact, without enough sleep, the brain cannot function properly, and this can impair one's ability to concentrate, think clearly, and process memories [6]. The recommended duration of sleep for adults (aged 18-64 years) is 7 to 9 hours [7]. Several studies have been conducted globally to examine the influence of the COVID-19 pandemic on sleep [8-10]. In Canada, Shillington et al. (2022) [8] assessed the sleep quality and quantity of Ontario adults during the initial phase of the pandemic (April-January 2020) and found that nearly two-thirds of the participants were “poor sleepers”. Fear, anxiety, and stress related to COVID-19 were found to be substantive self-reported contributors. Similarly, in Italy, Franceschini et al. (2020) [9] identified that, during the lockdown (from March 10 to May 4, 2020), factors such as job insecurities, fear of becoming infected with COVID-19, financial instabilities, escalated use of social media to seek information about the pandemic status and disrupted social relationships affected sleep quality and precipitated insomnia. In an Indian study that evaluated the sleep quality of 808 inhabitants between April 17 and May 24, 2020, more than half (57.2%) had poor sleep quality, and those with self-reported mental health deterioration were more likely to experience poor sleep quality [10]. Although this valuable study was the first to assess the sleep quality of citizens of India during the initial months of the pandemic, a fuller understanding of the sociodemographic, behavioral, and clinical predictors of sleep quality as well as identifying if differences exist throughout the country remains unknown. Furthermore, a specific focus on middle-aged adults who might be especially prone to lockdown-related challenges due to work demands, childcare requirements, and elder-care responsibilities is warranted [11].

## Materials and methods

Study design

This study presents the baseline sleep-related data from a larger and ongoing longitudinal study titled “Health Outcomes for Adults during and following the Covid-l9 PandEmic: The HOPE India Study”, which was modelled after and includes members from "The HOPE Study” from Canada [8]. The primary focus of these two longitudinal studies, from India and Canada, was to study the impact of the COVID-19 pandemic on lifestyle-related health behaviors and the overall well-being of adults. The specific objectives of "The HOPE Study” from India are to examine the lifestyle-related health behaviors (movement, diet) and overall well-being (including physical, mental health, and sleep quality) of adults (30-59 years) residing in parts of India during and after the stringent social distancing mandate of the COVID-19 pandemic. The present study reports the analyses of the baseline data that were collected while India experienced its stringent social distancing mandates (defined as the closing of both schools and bars, where alcohol is served in a particular state or union territory; October 2020-April 2021).

Participants 

Participants were recruited via a web-based survey. To be eligible for the study, participants needed to be: 1) residents of one of the three Indian states, one state each from north, central and south India, namely Madhya Pradesh, Kerala, or Delhi; 2) aged 30-59 years at baseline; 3) engaged in no international travel in the last two years; and 4) able to read and write in English, Malayalam, or Hindi.

Study duration and sample size

The present study was conducted between October 2020 and April 2021. The study included 694 participants. Since the goal was to include as many participants as possible, the sample size was not calculated at the outset for this sub-analysis, although this was done for the overarching India “HOPE Study”. However, the sample size was calculated retrospectively, assuming an 18% prevalence of sleep disturbance, a relative precision of 20%, and a non-response rate of 10%, resulting in a minimum sample size requirement of 550, which confirms that the power of the study was adequate [12]. Post hoc power calculations provided further confirmation (power=99%).

Study questionnaire

Sociodemographic, behavioral, and other background variables: The socio-demographic data collected included age, gender, the current address of residence (state, district, urban or rural area), marital status, per capita income, education, occupation, and the type of family (nuclear or joint family). Information collected on behavioral factors included current tobacco, alcohol use, and daily screen time. Anthropometric information such as self-reported height and weight, and information on history of diagnosis of COVID-19 were also collected.

The monthly per capita income cut-off of 3931 INR (USD$49.19) was used to determine upper and lower socio-economic status which is based on BG Prasad's socioeconomic scale of May 2021 [13].

Study tools

(a) PHQ-9 AND GAD-2 (Depression and Anxiety Measurement Tools): Participants were screened for depression using the Patient Health Questionnaire 2 (PHQ 2) and anxiety using the Generalized Anxiety Disorder 2 (GAD 2) [14-15]. The PHQ-2 enquires about the frequency of depressed mood over the past two weeks. The PHQ-2 includes the first two items of the PHQ-9. The PHQ-2 total score ranges from 0-6. A score of three or greater indicates major depressive disorder [14]. The GAD-2 is a brief and easy-to-perform initial screening tool for generalized anxiety disorder. A score of 3 points is the preferred cut-off for identifying possible cases in which further diagnostic evaluation for generalized anxiety disorder is warranted [15].

b) The Pittsburgh Sleep Quality Index (PSQI): The PSQI was utilized to assess sleep quality. PSQI is a self-rated instrument to assess sleep quality and screen for sleep disturbances over one month. There are 19 questions representing seven domains of sleep quality: sleep latency, sleep duration, subjective sleep quality, sleep efficiency, sleep disturbance, daytime dysfunction, and sleep medication use. The originators' scoring system recommended that the seven domains to be rated individually, then added together to produce a single "global" score with a potential range of 0 to 21 (zero indicating no difficulty with sleep and 21 indicating severe difficulties in all areas). A global score of more than 5 denotes a poor quality of sleep [16]. The PSQI tool has been validated for the Hindi and Malayalam-speaking population in India [16-17].

Data collection procedure

Survey forms were posted and shared on social media (e.g., Facebook, WhatsApp, Instagram, Twitter, and LinkedIn). The first page of the form included an informed consent page. Upon agreeing to participate in the study, interested participants were directed to the survey page. The above-noted tools (PHQ-2, GAD-2, and PSQI) were also incorporated into Microsoft survey forms to support their online completion. The links were first shared on social media platforms by the investigators to their primary contacts, who were requested to complete the survey, and share and disseminate the link as much as possible among their contacts (secondary contacts), thus maximizing the effect of snowball sampling. The data were collected using a self-reported questionnaire administered in English and local languages (Hindi for MP and Delhi; Malayalam for Kerala). The back translation of the questionnaire to English was done to ensure its linguistic validation in local languages (i.e., Hindi and Malayalam).

Ethical consideration

Ethical committee clearance was received vide Ref no.11/IEC/21/AIMS-08 from the Institutional Ethics Committee at Amala Institute of Medical Sciences, Thrissur, Kerala.

Statistical analysis

The data were analyzed using Excel 365 and SPSS version 24 (IBM Corp., Armonk, NY). Tableau Salesforce version 2021.4.3 (Tableau Software, Seattle, USA) was used to create the geo-mapping. Background information with categorical data were presented as frequencies and percentages. BMI was categorized according to the WHO standards (underweight <18.5, normal 18.5 - 24.99; overweight ≥ 25 -29.99; and obese ≥ 30).

Global PSQI scores were calculated per the tool's standard scoring guidelines and expressed as means and standard deviations. A pre-validated cut-off score of five or above five was used to indicate poorer sleep quality (sensitivity =89.6%, specificity =86.5%, kappa=0.75, p <0.001) [16,18]. A Chi-square test was used to determine the association between categorical variables. For regression analyses, those who received employment-related earnings were labelled “employed”, while the rest were classified as “others”, which included homemakers, students, retired from jobs, and pensioners. Univariable and multivariable logistic regression analyses were used to determine the predictors of poor sleep quality. Unadjusted and adjusted odds ratios were reported respectively. The variables which had p-value <0.25 in univariable analysis were included in the multivariable model. All tests were carried out with a 95% confidence interval and a significant p-value of ≤0.05.

## Results

The final sample consisted of 694 participants (374 females and 320 males). The mean (SD) age of the participants was 44 (9) years old, with an almost equal share of participants from across three strata of 10-year age groups (i.e., 30-39; 40-49; 50-59). Detailed background characteristics are mentioned in Table 1.

**Table 1:** Background characteristics of the participants (N = 694) Note: The monthly per capita income cut-off of 3931 INR (USD$49.19) was used to determine upper and lower socio-economic status which is based on BG Prasad socioeconomic scale of May 2021 [13].

Variables		n (%)
State	Madhya Pradesh	305(43.9)
	Delhi	46 (6.6)
	Kerala	343(49.5)
Residence	Rural	316(45.5)
	Urban	378(54.5)
Gender	Male	320(46.1)
	Female	374(53.9)
Age group (in years)	50-59	236(34)
	40-49	222(32)
	30-39	236(34)
Marital status	Married	607(87.4)
	Others	87(12.6)
Occupation	Professional	241(34.7)
	Semi-professional/clerk/shop owner farmer	120(17.3)
	Skilled	45(6.5)
	Unskilled	39(5.6)
	Unemployed	249(35.9)
Education	Professional degree	241(35.7)
	Graduate degree	215(30.9)
	Intermediate/diploma	92(13.3)
	High school	69(9.9)
	Middle school	43(6.2)
	Primary school	26(3.7)
	Illiterate	08(1.2)
Socio-Economic status*	Lower	250(39.2)
	Upper	388(60.8)
Family type	Joint family	266(38.3)
	Nuclear family	428(61.7)
BMI Categories	<18.5	26(3.7)
	18.5-24.9	322(46.4)
	25-29.9	260(37.5)
	≥30	86(12.4)
Screen time	≥3 Hours	259(37.3)
	<3 hours	435(62.7)
Current tobacco use	Yes	61(8.8)
	No	633(91.2)
Current alcohol use	No	86(87.6)
	Yes	608(12.4)
History of diagnosis of COVID 19 (Positive test result)	Absent	644(92.8)
	Present	50(7.2)
Depression	Screened negative	625(90.1)
	Screened positive	69(9.9)
Anxiety	Screened negative	614(88.5)
	Screened positive	80(11.5)
*N=638 because of missing data		

Sleep quality domains: A total of 59 out of 694 (8.5%) of the participants reported poor subjective sleep quality. About a quarter of the study, participants had a sleep latency of more than 30 minutes, with one-fourth of them having a latency of nearly an hour. Only 5% of the individuals consumed sleep medications, even though one-fifth of the participants had moderate to severely disrupted sleep (Table 2).

**Table 2:** Frequencies for PSQI among participants residing in three states of India. PSQI: Pittsburgh sleep quality index

Domain	Total Responses	Category and Frequency
Subjective sleep quality	694	Very good = 315
		Fairly good = 320
		Fairly bad = 57
		Very bad = 02
Sleep latency*	694	0 = 283
		1-2 = 208
		3-4 = 150
		5-6 = 53
Sleep disturbances*	694	0 = 132
		1-9 = 429
		10-18 = 122
		19-27 = 11
Use of sleep medication	694	Not during the past month = 552
		Less than once a week = 136
		Once or twice a week = 26
		Thrice or more times a week = 10
Daytime dysfunction*	694	0 = 487
		1-2 =167
		3-4= 35
		5-6 =5
Sleep duration	647	>7 hours =150
		7-6 hours = 138
		6-5 hours = 131
		<5 hours= 228
Habitual sleep efficiency (%)	647	>85 = 358
		75-84 = 77
		65-74 = 35
		<65 = 177
Global PSQI (Mean score=5.99±3.23, Range 0-17)	647	0-5 = 295
		6-10 = 293
		11-15 = 56
		16-21 = 03
* These categories are cumulative of sub-scores as per PSQI calculation guidelines		

**Figure 1:**
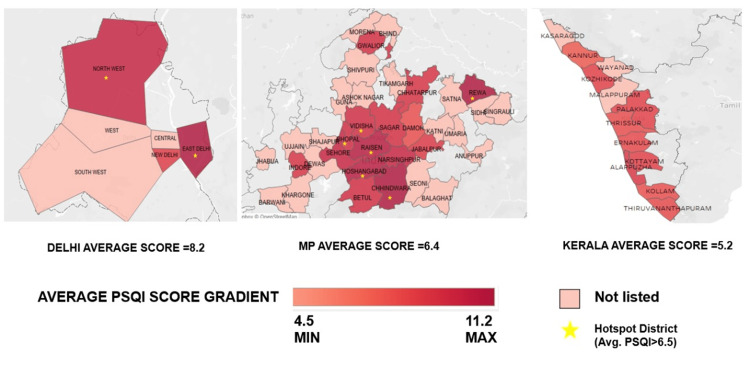
Geospatial map depicting the gradient of PSQI score among districts/divisions of three Indian states Geospatial map depicting the gradient of PSQI: Pittsburgh sleep quality index (PSQI) score among districts/divisions of three Indian states along with identified hotspot districts with poor sleep quality (PSQI >6.5). Figures show three Indian states with average PSQI grading ranging from 4.5 to 11.2. Hot spot districts (Average PSQI >6.5) are marked with a yellow star. Not listed are the districts with fewer than 5 participants.

Geolocation: A geospatial map depicting the gradient of PSQI scores among districts/divisions of three Indian states along with identified hotspot districts i.e. those with poor sleep quality (PSQI >6.5) is provided in Figure 1. The cut-off of 6.5 was set arbitrarily depending on clinical severity and was decided by the investigators in the absence of any previously published literature on such a cut-off. Each district is represented in the map as a cluster, and the spatial clustering of average PSQI scores is mapped at the district level. Only those districts with at least five participants were included in this analysis for meaningful results to be arrived at. Cluster PSQI values ranged from 4.5 to 11.2 on average. Delhi State had the highest average score (8.2), followed by Madhya Pradesh (6.4), while Kerala had the lowest score (5.2). Six districts in Madhya Pradesh (viz. Bhopal, Raisen, Hoshangabad, Chhindwara, Vidisha, and Rewa), two in Delhi (North West and East Districts), and none in Kerala were identified as hotspot districts.

Predictors of sleep quality: Univariable logistic regression showed that participants who belonged to Delhi and Madhya Pradesh (as against Kerala), resided in an urban area, were younger (30-39 years), belonged to the upper socioeconomic class, and screened positive for anxiety and depression had higher odds of having disturbed sleep (PSQI Score >5) as compared to their counterparts (Table 3).

**Table 3:** Socio-demographic, clinical, and contextual factors predicting the poor quality of sleep among the participants *Statistically significant = P<0.05. The results of the univariable logistic regression analysis are shown here. PSQI: Pittsburgh sleep quality index

Variable	Categories	PSQI score ≤5	PSQI score>5	Total	Odds ratio	Confidence interval	P value
State	Kerala	175(59.1)	121(40.9)	296(100)	Reference		
	Delhi	16(34.8)	30(64.2)	46(100)	1.099	0.568- 2.126	0.780
	Madhya Pradesh	104(34.1)	201(65.9)	305(100)	2.406	0.257-0.498	0.002*
Residence	Rural	141(51.1)	135(48.9)	276(100)	Reference		
	Urban	154(41.5)	217(58.5)	371(100)	1.472	1.076-2.014	0.016*
Gender	Female	160(46.3)	186(53.7)	346(100)	Reference		
	Male	135(44.9)	166(55.1)	301(100)	1.106	0.776-1.443	0.723
Age group (in years)	50-59	101(48.6)	107(51.4)	208(100)	Reference		
	40-49	106(50.5)	104(49.5)	210(100)	0.926	0.631- 1.359	0.695
	30-39	88(38.4)	141(61.6)	229(100)	1.512	1.034-2.213	0.033*
Marital status	Married	261(46.1)	305(53.9)	566(100)	Reference		
	Unmarried/Widow/Separated or divorced	33(41.3)	47(58.7)	80(100)	1.219	0.758-1.959	0.414
Occupation	Not employed currently	106(45.6)	126(54.3)	232(100)	Reference		
	Currently employed	189(45.5)	226(54.5)	415(100)	1.006	0.729-1.389	0.971
Education	Till higher secondary (12 years of schooling)	102(47.7)	112(52.3)	214(100)	Reference		
	Graduate & above	193(44.6)	240(55.4)	443(100)	1.132	0.185-1.573	0.458
Socio-economic status	Lower	122(52.8)	109(47.2)	231(100)	Reference		
	Upper	157(43.6)	203(56.3)	360(100)	1.447	1.039-2.017	0.029*
Type of Family	Joint family	119(47.2)	133(52.8)	252(100)	Reference		
	Nuclear family	176(44.6)	219(55.4)	395(100)	1.113	0.811- 1.529	0.507
BMI Category	<24.9	151(47.5)	167(52.5)	318(100)	Reference		
	≥25	144(43.8)	185(56.2)	329(100)	1.162	0.852-1.583	0.343
Screen time	<3 hours	189(47.8)	206(52.2)	395(100)	Reference		
	≥3 hours	106(42.1	146(57.9)	252(100)	1.264	0.919-1.738	0.150
History of diagnosis of COVID 19 (Positive test result)	Absent	276(46.2)	332(53.8)	598(100)	Reference		
	Present	19(38.8)	30(61.2)	49(100)	1.353	-0.294-0.899	0.320
Current tobacco use	No	274(46.7)	313(53.3)	587(100)	Reference		
	Yes	21(35)	39(65)	60(100)	1.626	0.934-2.831	0.086
Current alcohol use	No	249(45.8)	307(54.2)	566(100)	Reference		
	Yes	36 (44.4)	45(55.6)	81(100)	1.055	0.660-1.685	0.824
Depression	Screened negative	273(47.1)	307(52.9)	580(100)	Reference		
	Screened positive	22(32.8)	45(67.2)	67(100)	1.819	1.065-3.107	0.028*
Anxiety	Screened negative	275(48.2)	295(51.8)	570(100)	Reference		
	Screened positive	20(26)	57(74)	77(100)	2.657	1.556-4.537	<0.001*

In multivariable logistic regression analysis, the adjusted odds ratio remained significant for the state variable and the presence of anxiety, suggesting these two factors as independent predictors for poor sleep (Table 4). Participants from Delhi had a 33% (aOR=0.67, CI=0.32-2.37) lower likelihood of having disrupted sleep than those from Madhya Pradesh, while participants from Kerala had a 62 percent (adjusted odds ratio {aOR}=0.38, CI=0.25-0.53, P=0.000*) lower chance of having disturbed sleep than those from Madhya Pradesh. When compared to individuals who screened negative for anxiety, those who screened positive had a 2.4 times higher chance of experiencing disrupted sleep (aOR=2.4, CI=1.29-4.48, P=0.006*) (Table 4).

**Table 4:** Socio-demographic, clinical, and contextual factors predicting the poor quality of sleep among the participants The results of multivariable logistic regression analysis are shown here. *Significance =P<0.05

Variable	Categories	Adjusted Odds ratio	Confidence interval	P value
State	Madhya Pradesh	Reference		
	Delhi	0.672	0.329- 1.374	0.276
	Kerala	0.377	0.253-0.561	<0.001*
Residence	Rural	Reference		
	Urban	1.007	0.681-1.487	0.973
Age group (in years)	50-59	Reference		
	40-49	0.908	0.593-1.391	0.657
	30-39	1.264	0.829-1.926	0.267
Socio-economic status	Lower	Reference		
	Upper	1.311	0.916-1.878	0.139
Screen time	<3hours	Reference		
	≥3 hours	1.312	0.907-1.896	0.149
Current Tobacco use	No	Reference		
	Yes	1.247	0.674-2.309	0.482
Depression	Screened negative	Reference		
	Screened positive	1.139	0.600-2.163	0.691
Anxiety	Screened negative	Reference		
	Screened positive	2.400	1.285-4.484	0.006*

## Discussion

The purpose of this study was to document sleep quality and assess its sociodemographic, behavioral (i.e., tobacco use, alcohol use, and screen time), and mental-health-related indicators (i.e., anxiety and depression) in adults aged 30-59 years across three states of India, and to geo-locate state and district-level findings of sleep quality during the ongoing pandemic (October 2020 to April 2021). Participants’ mean global PSQI score was greater than five, indicating poor sleep quality. People residing in Madhya Pradesh were found to be the most affected with respect to sleep quality and disturbances. Anxiety was found to be an important independent predictor of poor sleep quality.

In India, community-based research on sleep disruptions has revealed a significant hidden burden of the condition [12,18]. The ongoing pandemic, as well as its impact on lifestyle, has not only brought these difficulties to light but has also exacerbated the problem. This was evident from a survey conducted in India before the pandemic, where disrupted sleep was shown to be prevalent in only 18% of the population [12]. An Indian study among adults during the COVID-19 pandemic reported high PSQI scores, with 57.2% of the respondents having poor sleep quality [10]. Similar findings were reported during the early phase of COVID on sleep quality in an article from the HOPE Canada project, with over two-thirds of individuals identified as having poor sleep quality [8]. A systematic review on sleep problems during the COVID-19 pandemic combined six studies from the general population (n=4722) and reported the pooled prevalence of disturbed sleep (using PSQI) to be 37.9% with an average PSQI score of 6 [19]. In the present study, more than half of the respondents reported sleeping for less than 6 hours a day. This is well below the recommended 7-8 hours of sleep for an adult [7]. Findings from the current study are about one hour less than what was reported by another Indian study during the pandemic, where participants’ self-reported mean sleep, at 6.9 hours, was nearly reaching the lower end of sleep duration guidelines [10].

Normal documented adult sleep efficiency is 85-90% [20].In the present study, about 33% of participants had a sleep efficiency of less than 75%, which is considered poor sleep efficiency and indicative of insomnia [20]. We also found that around 29% of the study participants had a sleep latency of more than 30 minutes, which is well above the normal adult sleep latency of 10-20 minutes [21]. Sleep latency of more than 20 minutes falls under the category of insomnia. In a large (n=72,262) pre-pandemic study conducted in India, Pengpid et al. (2021) [22] found the prevalence of insomnia to be 12.7% among adults. Certainly, the problem has increased during the pandemic. This high prevalence of insomnia during the pandemic, now referred to by some researchers as COVID-somnia, can be attributed to fear of dying from the disease and/or the result of drastic lifestyle adjustments due to the pandemic [23].

Compared to residents of Madhya Pradesh, Delhi, and Kerala residents experienced 33% and 62% lower chances of sleep disturbance respectively, and the results for Kerala were statistically significant. The maximum number of hotspot districts with severe sleep disturbances were from Madhya Pradesh. The probable explanation is that during this period, Kerala recorded one of the least case fatality rates in the country, well below the national average of 1.2% [24], whereas, case fatality rates of Madhya Pradesh (1.3%) and Delhi (1.4%) were above the national average [25]. These findings might be linked to the high fear of dying in the two states compared to Kerala. To potentiate this hypothesis, we also found that the proportion of anxiety and depression was also lowest in Kerala compared to the overall prevalence of 11.5% and 10.5%, respectively. Additionally, Kerala has a better overall health service than other included states, therefore, improved health care may possibly be a likely factor [26].

It has been known that depression and anxiety can lead to sleep disorders [27]. Also, the implications of the COVID-19 pandemic on mental health are well documented [28]. The current study also showed that people who screened positive for depression and anxiety had a higher likelihood of having sleep problems. After adjustment for other variables anxiety emerged as a significant predictor of poor sleep quality. Studies conducted in India and abroad also corroborated these findings [9,12,20].

In the present study, sleep disturbances were more prominent among those who were single, separated/widowed/widower/divorced, compared to those who were married, though not statistically significant. The probable reason for this finding could be the spousal emotional support to handle the anxiety, fear of dying, and coping with the loss [29]. In the present study, those with a BMI of more than 25 had a slightly higher prevalence of disturbed sleep compared to those with a BMI of less than 25, but this was not statistically significant. Studies in the past have linked disturbed sleep to higher BMI, being overweight, or being obese [22].

In the present study, although not statistically significant, those who had suffered from COVID-19 (tested positive) had a higher prevalence of disturbed sleep compared to those without a history of being tested COVID-positive. Similar findings were reported by Jahrami et al. 2021 [30]. This might be attributed to the direct pathological effect of COVID-19 and the psychological impact of the disease [30].

Limitations

Because it was a self-reported online survey, people without access to electronic devices or internet access may have been missed, which is a limitation of most online surveys. Furthermore, the coverage may have been limited because the link to the questionnaire was shared mainly through the primary and secondary contacts of investigators belonging to India. Also, the impact of job and financial losses on sleep quality during the studied stage of the pandemic was not thoroughly investigated. Since this was a cross-sectional analysis, reverse causality cannot be ruled out.

## Conclusions

Sleep quality was poor among Indian adults during the COVID-19 epidemic (October 2020-April 2021), as evidenced by the global PSQI scores, and the fact that more than half of the participants had poor sleep quality. Anxiety emerged as an important predictor of poor-quality sleep. In terms of sleep disruptions, those belonging to Madhya Pradesh were the most affected, followed by those from Kerala and Delhi, highlighting the existence of state-level differences within the country.
